# Use of Blood Donor Screening to Monitor Prevalence of HIV and Hepatitis B and C Viruses, South Africa

**DOI:** 10.3201/eid2309.161594

**Published:** 2017-09

**Authors:** Marion Vermeulen, Ronel Swanevelder, Dhuly Chowdhury, Charlotte Ingram, Ravi Reddy, Evan M. Bloch, Brian S. Custer, Edward L. Murphy, for the NHLBI Recipient Epidemiology and Donor evaluation Study-III (REDS-III) International Component

**Affiliations:** South African National Blood Service, Johannesburg, South Africa (M. Vermeulen, R. Swanevelder, C. Ingram, R. Reddy);; RTI International, Rockville, Maryland, USA (D. Chowdhury);; Johns Hopkins University School of Medicine, Baltimore, Maryland, USA (E.M. Bloch);; Blood Systems Research Institute, San Francisco, California, USA (B.S. Custer, E.L. Murphy);; University of California, San Francisco (E.L. Murphy)

**Keywords:** HIV, hepatitis B virus, hepatitis C virus, prevalence, South Africa, blood donors, viruses, hepatitis

## Abstract

Among 397,640 first-time blood donors screened in South Africa during 2012–2015, HIV prevalence was 1.13%, hepatitis B virus prevalence 0.66%, and hepatitis C virus prevalence 0.03%. Findings of note were a high HIV prevalence in Mpumalanga Province and the near absence of hepatitis C virus nationwide.

South Africa has one of the largest HIV epidemics in the world. HIV prevalence is 18.8% among those 15–49 years of age, and estimated HIV incidence in sexually active persons is 1.21/100 person-years for men and 2.28/100 person-years for women (*1*,*2*). Chronic hepatitis B virus (HBV) infection is also common; among young adults, hepatitis B surface antigen (HBsAg) prevalence is ≈4%, and universal HBV vaccination of infants was introduced in 1995 (*3*). Other than in an outdated study that found PCR-positive hepatitis C virus (HCV) in 0.05% of blood donors (*4*), the prevalence of HCV infection in South Africa is poorly described but is probably lower than in other countries in Africa (*5*). Recent published data on the prevalence of HIV, HBV, and HCV among blood donors in South Africa are scant (*6*,*7*). We assessed prevalence of these viruses by demographic and geographic characteristics to inform donor-selection criteria and to aid public health surveillance.

## The Study

We included all eligible first-time blood donors at South African National Blood Service (SANBS) facilities for January 2012–September 2015, covering all provinces except Western Cape Province. We excluded those deferred from donation because of risk behaviors or poor health. 

We screened blood donations individually for HIV RNA, HCV RNA, and HBV DNA by using the Procleix Ultrio Plus assay (Grifols, Barcelona, Spain) and serologically for HIV antibodies, HCV antibodies, and HBsAg by using Abbott Prism ChLia (Abbott, Delkenheim, Germany). We further tested serologic repeat–reactive but nucleic acid testing (NAT)–negative donations by using supplemental assays: HIV Western blot (Bio-Rad, Hercules, CA, USA); HCV InnoLIA (Innogenetics, Ghant, Belgium); or HBsAg neutralization (Roche, Pleasanton, CA, USA).

We calculated prevalences and derived odds ratios (ORs) and 95% CIs for associations from multivariable logistic regression by using SAS/STAT 9.4 (SAS Institute, Inc., Cary, NC, USA). Because of statistically significant interactions between sex and age and between sex and race (Technical Appendix 1), we built separate models for male and female donors.

During January 2012–September 2015, a total of 3,075,422 blood donations were made at SANBS facilities from repeat donors; 397,640 (13%) donations were from first-time donors, who were predominantly young and equally distributed by sex (Table). Approximately half of donors were black, one third white, and the remainder of Asian; South African Colored (SAC) (an admixed group made up of 5 source populations [African Khoisan, African Bantu, European, South Asian, and East Asian]); or unknown race/ethnicity.

**Table:** Prevalence of HIV, HBV, and HCV, by demographic characteristics, among persons making blood donations through the South African National Blood Service, January 2012–September 2015*

Characteristic	No. first-time donors	No. (%)		
		HIV-positive	HBV-positive	HCV-positive
Overall	397,640	4,481 (1.13)	2,638 (0.66)	125 (0.03)
Age group, y				
<20	185,983	1,139 (0.61)	382 (0.21)	6 (0.00)
20–29	103,373	1,702 (1.65)	999 (0.97)	39 (0.04)
30–39	55,420	1,038 (1.87)	721 (1.30)	17 (0.03)
40–49	33,330	440 (1.32)	366 (1.10)	21 (0.06)
50–59	16,518	146 (0.88)	151 (0.91)	31 (0.19)
>60	3,016	16 (0.53)	19 (0.63)	11 (0.36)
Sex				
M	177,729	1,396 (0.79)	1,635 (0.92)	77 (0.04)
F	219,903	3,085 (1.40)	1,003 (0.46)	48 (0.02)
Race/ethnicity†				
Black	211,722	4,204 (1.99)	2,355 (1.11)	62 (0.03)
White	122,894	74 (0.06)	80 (0.07)	43 (0.03)
Asian	28,428	28 (0.10)	41 (0.14)	11 (0.04)
SAC	20,246	98 (0.48)	99 (0.49)	5 (0.02)
Unknown	14,350	77 (0.54)	63 (0.44)	4 (0.03)
Province				
Eastern Cape	37,055	365 (0.99)	315 (0.85)	4 (0.01)
Free State	20,759	241 (1.16)	68 (0.33)	3 (0.01)
Gauteng	175,623	1,774 (1.01)	967 (0.55)	77 (0.04)
KwaZulu-Natal	80,111	918 (1.15)	728 (0.91)	14 (0.02)
Limpopo	15,661	159 (1.02)	113 (0.72)	7 (0.04)
Mpumalanga	35,720	779 (2.18)	305 (0.85)	8 (0.02)
Northwest	19,205	124 (0.65)	65 (0.34)	7 (0.04)
Northern Cape	10,333	74 (0.72)	57 (0.55)	3 (0.03)

*3,173 donors had missing information on province. HBC, hepatitis B virus; HCV, hepatitis C virus; SAC, South African Colored. †The Department of Home Affairs in South Africa classifies the South Africa population into 4 race groups: African, Indian, White, and Coloured. The SAC population is an admixed group made up of 5 source populations (African Khoisan, African Bantu, European, South Asian, and East Asian) dating back to slavery and the early settlers.

A total of 4,481 (1.13%) first-time donors were classified as HIV positive. Prevalence was highest (1.3%–1.9%) among persons 20–49 years of age, higher among female (1.4%) than male (0.8%) donors, and higher among those of black race/ethnicity (2.0%) than other races/ethnicities (Table). In logistic regression models (Technical Appendix 2), HIV infection was more strongly associated with older age among male donors than among female donors and more strongly with black and unknown race/ethnicity among female donors than among male donors (Technical Appendix 1). We observed a significant association between HIV and HBV infection in both sexes and a stronger association between HIV and HCV infection in female donors only. Compared with Gauteng Provence, HIV infection was associated with donation in Mpumalanga, KwaZulu-Natal, and Free State provinces for both sexes and with Eastern Cape Province for female donors and Northern Cape Province for male donors (Figure).

**Figure:**
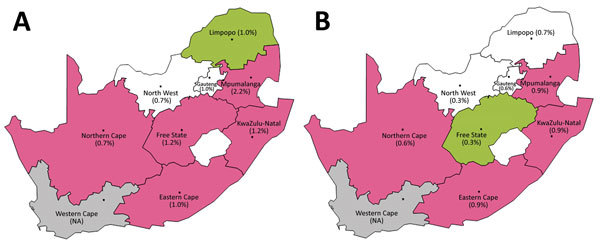
Overall prevalence of HIV (A) and hepatitis B virus (B) in South Africa, by province, among persons making blood donations through the South African National Blood Service, January 2012–September 2015. Pink indicates a significantly higher odds ratio and green indicates a lower odds ratio compared with Gauteng Province (Johannesburg region) and adjusting for other factors. Unadjusted prevalences are shown in parentheses. NA, not applicable.

The 1.13% HIV prevalence among first-time blood donors in South Africa is much higher than that for high-income countries but lower than for many countries in sub-Saharan Africa, where HIV prevalence ranges from 3% to 5% (*8*). HIV prevalence among donors was substantially lower than that among the general adult population of South Africa (estimated at 18.8%), but similar demographic associations were observed (*1*,*2*). Geographic distributions of HIV infection were also generally similar to national data, although we found higher adjusted odds for HIV infection in Mpumalanga Province compared with KwaZulu-Natal Province (*1*). Incorporation of blood donor prevalence and incidence data might help to refine statistical models of the HIV epidemic, which have not performed well in some subgroups (*2*,*9*). In addition, blood bank testing for HIV includes men and older persons, who are not well-represented in current surveillance strategies (*10*).

A total of 2,638 (0.66%) first-time donors were classified as HBV-positive. HBV prevalence was 0.9%–1.3% among those 30–49 years of age, and only 0.2% among those <20 years of age (Table). HBV prevalence was 0.9% among male donors versus 0.5% among female donors, 1.1% among blacks, 0.5% among persons of SAC race/ethnicity, and 0.1% among whites. In the logistic regression models (Technical Appendix 2), HBV infection was more strongly associated with older age among men than among women and had a geographic distribution slightly different from that of HIV.

The HBV prevalence of 0.66% was substantially less than the median of 4.35% for all countries in Africa; however, lack of confirmatory testing might inflate the proportion for all of Africa (*11*). In our study, a 5-fold lower prevalence among donors <20 years of age compared with those 20–29 years of age is consistent with the implementation of HBV vaccination of infants in South Africa in 1995 and could be used to estimate vaccination coverage (*3*). Male donors appear to be at higher risk for chronic HBV infection, as reported in the United States (*12*).

Only 125 (0.03%) donors were confirmed positive for HCV infection. HCV prevalence was highest (0.4%) among those >60 years of age (0.04% among men, 0.02% among women) (Table). We observed little difference in HCV prevalence by race/ethnicity. In logistic regression models, HCV infection was associated with older age and with HIV co-infection among women only (Technical Appendix 2). Among men only, HCV was inversely associated with blood donation in Eastern Cape and KwaZulu-Natal Provinces.

Contrary to some reports, which included small studies and those lacking confirmatory testing (*13*), HCV infection appears to be rare among South Africa blood donors and, by extrapolation, its general population. The 0.03% blood donor prevalence we found is consistent with an older study (*4*) and much lower than the median of 0.86% for other countries in Africa (*11*). Reasons for this low prevalence are unclear but likely relate to the relative absence of injection drug use or other parenteral risk factors for HCV transmission. Further study of why South Africa has lower HCV prevalence than many countries in the world is warranted. One clue might be the predominance of infection among older and male persons, suggesting a possible birth cohort effect related to historical parenteral exposures (*14*).

## Conclusions

Our study attests to the success of blood donor selection and screening: HIV prevalence was ≈18-fold lower and HBV prevalence 5-fold lower than that of the general population of South Africa. This difference is attributable to selection of low-risk and healthy donors and underrepresentation of blacks among blood donors. These biases need to be accounted for in extrapolating directly to the general population, but comparisons between donor subgroups or periods might still mirror population data. Prevalent infections in donors are detected by serologic testing, and blood products are discarded accordingly. To mitigate the risk posed by seronegative window period infections, SANBS performs routine individual donation NAT. This parallel serology and NAT testing has generated substantial data on HIV and HBV incidence, further contributing to public health surveillance (*6*).

Technical Appendix 1 Interaction effects of age and sex and race/ethnicity and sex in the multivariate logistic regression model for HIV infection.

Technical Appendix 2 Logistic regression models for HIV, hepatitis B virus, and hepatitis C virus infection, by sex.

## References

[1] Zuma K, Shisana O, Rehle TM, Simbayi LC, Jooste S, Zungu N, et al. New insights into HIV epidemic in South Africa: key findings from the National HIV Prevalence, Incidence and Behaviour Survey, 2012. Afr J AIDS Res. 2016;15:67–75. 10.2989/16085906.2016.1153491 27002359

[2] Eaton JW, Bacaër N, Bershteyn A, Cambiano V, Cori A, Dorrington RE, et al. Assessment of epidemic projections using recent HIV survey data in South Africa: a validation analysis of ten mathematical models of HIV epidemiology in the antiretroviral therapy era. Lancet Glob Health. 2015;3:e598–608. 10.1016/S2214-109X(15)00080-7 26385301

[3] Amponsah-Dacosta E, Lebelo RL, Rakgole JN, Burnett RJ, Selabe SG, Mphahlele MJ. Evidence for a change in the epidemiology of hepatitis B virus infection after nearly two decades of universal hepatitis B vaccination in South Africa. J Med Virol. 2014;86:918–24. 10.1002/jmv.23910 24615635

[4] Tucker TJ, Voigt M, Bird A, Robson S, Gibbs B, Kannemeyer J, et al. Hepatitis C virus infection rate in volunteer blood donors from the Western Cape—comparison of screening tests and PCR. S Afr Med J. 1997;87:603–5. 9254818

[5] Rao VB, Johari N, du Cros P, Messina J, Ford N, Cooke GS. Hepatitis C seroprevalence and HIV co-infection in sub-Saharan Africa: a systematic review and meta-analysis. Lancet Infect Dis. 2015;15:819–24. 10.1016/S1473-3099(15)00006-7 25957078

[6] Bruhn R, Lelie N, Custer B, Busch M, Kleinman S; International NAT Study Group. Prevalence of human immunodeficiency virus RNA and antibody in first-time, lapsed, and repeat blood donations across five international regions and relative efficacy of alternative screening scenarios. Transfusion. 2013;53:2399–412. 10.1111/trf.12299 23782054

[7] Vermeulen M, Lelie N, Sykes W, Crookes R, Swanevelder J, Gaggia L, et al. Impact of individual-donation nucleic acid testing on risk of human immunodeficiency virus, hepatitis B virus, and hepatitis C virus transmission by blood transfusion in South Africa. Transfusion. 2009;49:1115–25. 10.1111/j.1537-2995.2009.02110.x 19309474

[8] Bloch EM, Vermeulen M, Murphy E. Blood transfusion safety in Africa: a literature review of infectious disease and organizational challenges. Transfus Med Rev. 2012;26:164–80. 10.1016/j.tmrv.2011.07.006 21872426 PMC3668661

[9] Rehle T, Johnson L, Hallett T, Mahy M, Kim A, Odido H, et al. A comparison of South African national HIV incidence estimates: a critical appraisal of different methods. PLoS One. 2015;10:e0133255. 10.1371/journal.pone.0133255 26230949 PMC4521952

[10] Johnson LF, Rehle TM, Jooste S, Bekker LG. Rates of HIV testing and diagnosis in South Africa: successes and challenges. AIDS. 2015;29:1401–9. 10.1097/QAD.0000000000000721 26091299

[11] Apata IW, Averhoff F, Pitman J, Bjork A, Yu J, Amin NA, et al.; Centers for Disease Control and Prevention (CDC). Progress toward prevention of transfusion-transmitted hepatitis B and hepatitis C infection—sub-Saharan Africa, 2000-2011. MMWR Morb Mortal Wkly Rep. 2014;63:613–9. 25055184 PMC5779426

[12] Custer B, Kessler D, Vahidnia F, Leparc G, Krysztof DE, Shaz B, et al.; NHLBI Retrovirus Epidemiology Donor Study-II (REDS-II). Risk factors for retrovirus and hepatitis virus infections in accepted blood donors. Transfusion. 2015;55:1098–107. 10.1111/trf.12951 25470984 PMC4428964

[13] Gower E, Estes C, Blach S, Razavi-Shearer K, Razavi H. Global epidemiology and genotype distribution of the hepatitis C virus infection. J Hepatol. 2014;61(Suppl):S45–57. 10.1016/j.jhep.2014.07.027 25086286

[14] Mohd Hanafiah K, Groeger J, Flaxman AD, Wiersma ST. Global epidemiology of hepatitis C virus infection: new estimates of age-specific antibody to HCV seroprevalence. Hepatology. 2013;57:1333–42. 10.1002/hep.26141 23172780

