# Supplementary material for: Use of Blood Donor Screening to Monitor Prevalence of HIV and Hepatitis B and C Viruses, South Africa
Source: Emerg Infect Dis. 2017 Sep;23(9):1560–3. doi: 10.3201/eid2309.161594 (PMC5572879; doi:10.3201/eid2309.161594)
Supplement: Technical Appendix 1 — Interaction effects of age and sex and race/ethnicity and sex in the multivariate logistic regression model for HIV infection. [file 16-1594-Techapp-s1.pdf]

Article DOI: https://doi.org/10.3201/eid2309.161594

Use of Blood Donor Screening to Monitor Prevalence of HIV and Hepatitis B and C Viruses, South Africa

Technical Appendix 1

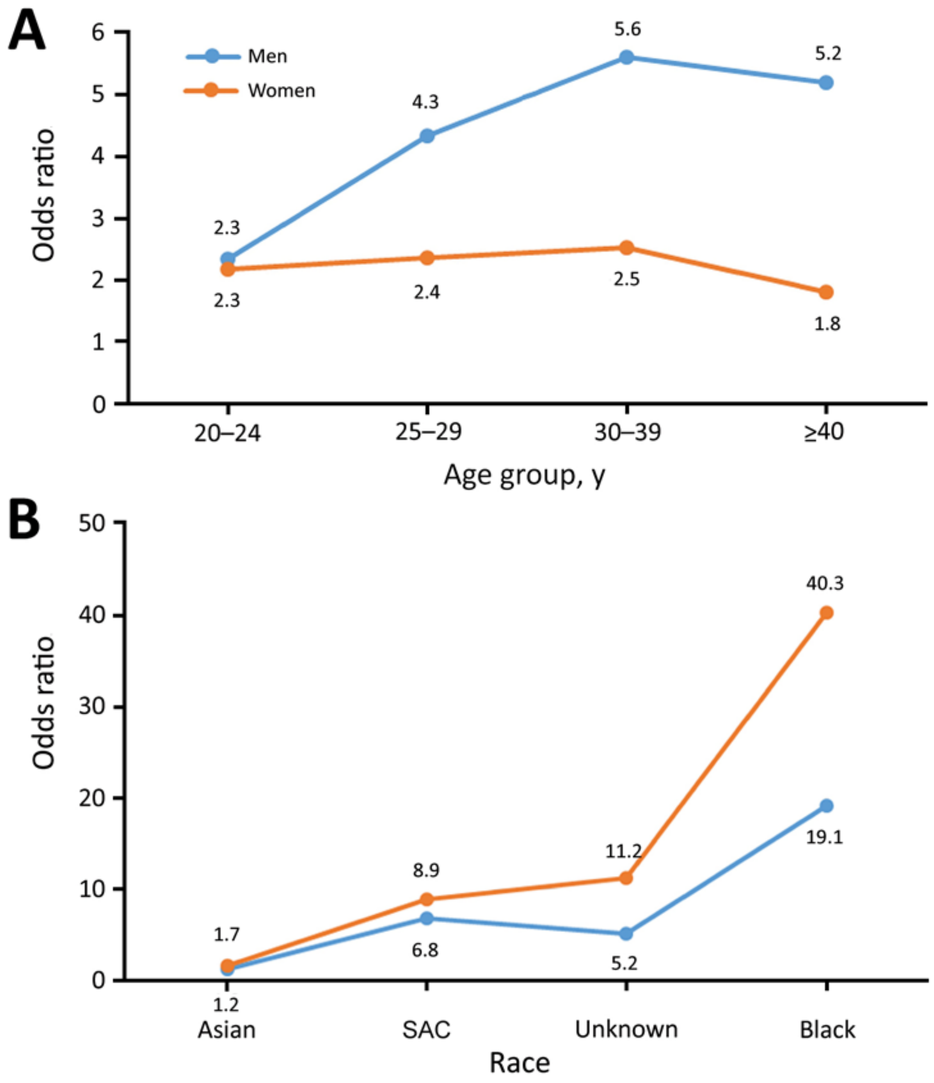

Technical Appendix 1 Figure. Interaction effects of age and sex (A) and race/ethnicity and sex (B) in the multivariate logistic regression model for HIV Infection. Adjusted odds ratios for each age or racial group compared with the reference group (age <20 years or white race) are graphed by sex. SAC, South African colored.
